# Conformational Motions and Functionally Key Residues for Vitamin B12 Transporter BtuCD–BtuF Revealed by Elastic Network Model with a Function-Related Internal Coordinate

**DOI:** 10.3390/ijms160817933

**Published:** 2015-08-04

**Authors:** Ji-Guo Su, Xiao Zhang, Shu-Xin Zhao, Xing-Yuan Li, Yan-Xue Hou, Yi-Dong Wu, Jian-Zhuo Zhu, Hai-Long An

**Affiliations:** 1 College of Science, Yanshan University, Qinhuangdao 066004, China; E-Mails: xiaozhangysuniv@163.com (X.Z.); zsx360@yeah.net (S.-X.Z.); lxy@ysu.edu.cn (X.-Y.L.); houyanxue@ysu.edu.cn (Y.-X.H.); wuyidong@ysu.edu.cn (Y.-D.W.); zhujz@ysu.edu.cn (J.-Z.Z.); 2 Key Laboratory of Molecular Biophysics, Institute of Biophysics, Hebei University of Technology, Tianjin 300130, China

**Keywords:** ABC transporter, BtuCD–BtuF, functional motions, key residues, elastic network model, internal coordinate, normal mode analysis, perturbation method

## Abstract

BtuCD–BtuF from *Escherichia coli* is a binding protein-dependent adenosine triphosphate (ATP)-binding cassette (ABC) transporter system that uses the energy of ATP hydrolysis to transmit vitamin B12 across cellular membranes. Experimental studies have showed that during the transport cycle, the transporter undergoes conformational transitions between the “inward-facing” and “outward-facing” states, which results in the open–closed motions of the cytoplasmic gate of the transport channel. The opening–closing of the channel gate play critical roles for the function of the transporter, which enables the substrate vitamin B12 to be translocated into the cell. In the present work, the extent of opening of the cytoplasmic gate was chosen as a function-related internal coordinate. Then the mean-square fluctuation of the internal coordinate, as well as the cross-correlation between the displacement of the internal coordinate and the movement of each residue in the protein, were calculated based on the normal mode analysis of the elastic network model to analyze the function-related motions encoded in the structure of the system. In addition, the key residues important for the functional motions of the transporter were predicted by using a perturbation method. In order to facilitate the calculations, the internal coordinate was introduced as one of the axes of the coordinate space and the conventional Cartesian coordinate space was transformed into the internal/Cartesian space with linear approximation. All the calculations were carried out in this internal/Cartesian space. Our method can successfully identify the functional motions and key residues for the transporter BtuCD–BtuF, which are well consistent with the experimental observations.

## 1. Introduction

Adenosine triphosphate (ATP)-binding cassette (ABC) transporters form a large family of proteins that use energy from ATP to power the transport of various substrates across biological membranes [1,2,3]. ABC transporters have been found both in prokaryotes and eukaryotes, which play important roles in nutrient uptake, toxin export, antigen processing and osmotic regulation [1,4,5]. Many human diseases, such as cystic fibrosis, Alzheimer’s disease, Stargardt disease, age-related macular degeneration, adrenoleukodystrophy, Tangier disease and obstetric cholestasis, have been linked to defects of ABC transporters [1,6,7,8,9,10]. The investigation of the molecular mechanism of ABC transporters is not only helpful for our understanding of many biological processes, but also has potential applications in the treatment of these related diseases.

**Figure 1:**
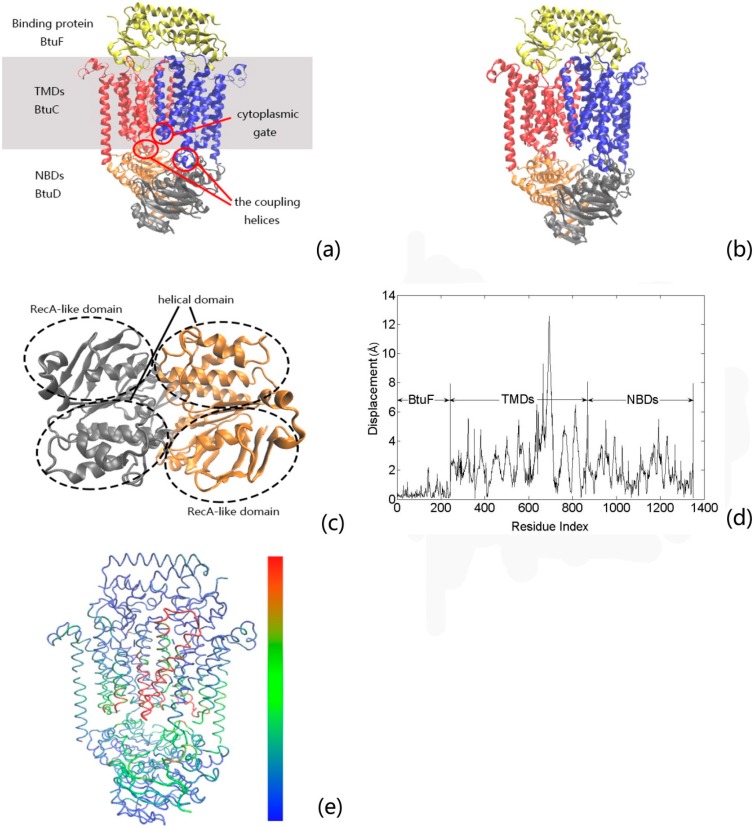
The tertiary structure of BtuCD–BtuF complex at the nucleotide-bound intermediate state (protein data bank (PDB) code: 4FI3) (**a**) and the apo complex state (PDB code: 4DBL) (**b**). BtuCD consists of two transmembrane domains (TMDs, *i.e.*, BtuC subunits) and two cytoplasmic nucleotide-binding domains (NBDs, *i.e.*, BtuD subunits). BtuF binds to the periplasmic side of BtuCD. The TMDs of BtuCD form the substrate translocation channel, whose cytoplasmic gate is marked by red circle in panel (**a**). In this study, the extent of opening of the cytoplasmic gate is chosen as the internal coordinate that related to the channel-gating function of the protein. The coupling helices that connect TMDs and NBDs are also marked by red circles in the figure; The top view of NBDs is displayed in panel (**c**), in which the helical and RecA-like domains are highlighted by dotted circles; The residue displacements between the two states of BtuCD–BtuF are displayed (**d**); In order to intuitively display the calculation results, the residue displacements are also mapped onto the protein structure (**e**), where the yellow-red color indicates larger residue displacements and the blue color denotes lower displacements.

BtuCD–BtuF is a type II ABC transporter system that uses the energy of ATP to translocate vitamin B12 from the periplasm to the cytoplasm of *Escherichia coli* [4,11,12]. The crystal structures of full-length BtuCD–BtuF complex at two distinct states have been determined by X-ray crystallography [13,14]. One is the nucleotide-bound intermediate state (protein data bank (PDB) code: 4FI3) and the other is the apo BtuCD–BtuF complex (PDB code: 4DBL), as shown in Figure 1a,b, respectively. BtuF is the substrate binding protein that binds vitamin B12 with high affinity and specificity in the bacterial periplasm, and then delivers it to the transporter [15,16]. BtuCD is the ABC transporter that mediates the uptake of the substrate into the cell, which consists of two transmembrane domains (TMDs) (*i.e*., BtuC subunits) and two cytoplasmic nucleotide-binding domains (NBDs) (*i.e*., BtuD subunits), as shown in Figure 1a. The dimeric TMDs form the substrate translocation channel, whose cytoplasmic gate (around BtuC residue 143) controls the translocation of substrate into the cell [17]. The two NBDs of the transporter are responsible for ATP binding and hydrolysis. Each NBD monomer consists of a helical domain and a RecA-like domain, as shown in Figure 1c. The TMDs and NBDs are connected through two coupling helices, as shown in Figure 1a.

Based on the various structures of BtuCD–BtuF complex at different states, a peristaltic transport mechanism has been proposed by Korkhov *et al.* for this transporter [13]. The transport cycle is triggered by the docking of BtuF to the periplasmic face of BtuCD. Then, the two NBD monomers move together and bind two ATP molecules. The closure of the NBD dimer results in the shortening of the distance between the two TMDs–NBDs coupling helices and also induces the conformational rearrangements in the TMDs, which trap the substrate B12 in the translocation cavity. After the hydrolysis of ATP and release of the hydrolysis products, the closed NBD dimer will open. The opening of NBD dimer pulls the two coupling helices to swing away, which then drives the cytoplasmic gate in TMDs to open. After the opening of the cytoplasmic gate, the vitamin B12 was squeezed out of the cavity in TMDs and ejected into the cytoplasm. Then, the transporter arrives at the apo BtuCD–BtuF complex state. After the release of BtuF, the transporter returns to the initial conformation and a new transport cycle will be allowed. Experimental studies have shown that during the transport cycle, the NBDs and the coupling helices undergo large-scale rigid body movements, as in other ABC transporters. However, the motions of TMDs and BtuF resemble peristalsis rather than large-scale rigid body movements, which is different from that of type I ABC transporter [13]. To illustrate this point, the residue displacements between the two published structures of the full-length BtuCD–BtuF at the nucleotide-bound intermediate (Figure 1a) and the apo complex (Figure 1b) states were calculated. The calculation result is displayed in Figure 1d, and the residue displacements are mapped onto the protein structure in Figure 1e. From these figures, it is found that the NBDs and the cytoplasmic side of TMDs have relatively large displacements, whereas the displacements of the residues in BtuF and the periplasmic side of TMDs are not distinct.

As discussed above, the BtuCD–BtuF system undergoes functional domain motions during the substrate transport cycle and there exist long-range allosteric couplings between different domains of the protein. Several questions are raised needing to be answered. Whether are these domain motions and long-range allosteric communication encoded in the structural topology of the transporter? How can we effectively extract the functional motions from the tertiary structure of the protein? It is believed that the collective motions in proteins usually involve some key residues that mediate the conformational changes between spatially separated subparts of the proteins [18,19]. How can we identify these functionally key residues in the structure of the transporter?

Normal mode analysis (NMA) of the elastic network model (ENM) is a simple yet effective method to reveal the motion modes encoded in protein tertiary structure, which has been largely used to explore the collective domain motions and identify the function-relevant key residues of proteins [20,21,22,23,24,25,26,27,28,29,30,31]. However, the motion modes revealed by the conventional NMA approach do not necessarily correspond to a specific function of proteins. Hub and de Groot introduced a quantity that is directly relevant to a specific protein function, and then proposed an analysis method to detect the collective motion that is maximally correlated to that quantity [32]. Capozzi *et al.* introduced variables of inter-helical angles, combined with NMA, to analyze the fluctuation dynamics of the helices in the EF-hand proteins [33]. Motivated by these approaches, in the present work, a new method was proposed to analyze the channel-gating function of the transporter and identify the functionally key residues in the protein structure. As described above, the open-closed motions of the cytoplasmic channel gate control the translocation of the substrate into the cell. In this study, the extent of opening of the cytoplasmic gate was chosen as an internal coordinate that is relevant to the channel-gating function of the transporter. Then, the mean-square fluctuation of the internal coordinate (MSFIC) for each normal mode was calculated to evaluate the contribution of each mode to the channel-gating motions, and the modes with relative large MSFIC value are identified as the function-related motion modes. Moreover, the cross-correlation between the displacement of the internal coordinate and the movement of each residue in the protein was computed to reveal the functional motions of the transporter. In addition, the functionally key residues were identified by using a perturbation method, in which the residues whose perturbation largely influences the fluctuation along the internal coordinate were considered as the key residues. In order to facilitate these calculations, the internal coordinate was introduced as one of the axes of the coordinate space and the conventional Cartesian coordinate space was transformed into the internal/Cartesian space with linear approximation. All the calculations were carried out in this internal/Cartesian space. Using this method, the functional motions and key residues for the transporter BtuCD–BtuF were investigated and compared with the experimental observations.

## 2. Results and Discussion

### 2.1. The Contribution of Each Motion Modes to the Channel-Gating Function of BtuCD–BtuF

In the present work, the extent of opening of the cytoplasmic gate was chosen as the internal coordinate that is related to the channel-gating function of BtuCD–BtuF. The extent of opening of the gate is defined as the average distance between residues located on the opposite sides of the cytoplasmic gate. X-ray crystallographic studies have shown that the residues around 143 from the two monomers of TMD form the cytoplasmic gate [17]. In the present work, the crystal structure of BtuCD–BtuF at the nucleotide-bound intermediate state (PDB code: 4FI3) was used for our calculation. Four residues around the cytoplasmic gate, *i.e.*, Ser143, Arg144, Leu147 and Ala148, in each monomer were chosen, and the average distance between the residue pairs formed by these residues from the two monomers, *i.e.*, Ser143–Ser143, Arg144–Arg144, Leu147–Leu147 and Ala148–Ala148, was calculated to serve as the functional internal coordinate.

**Figure 2:**
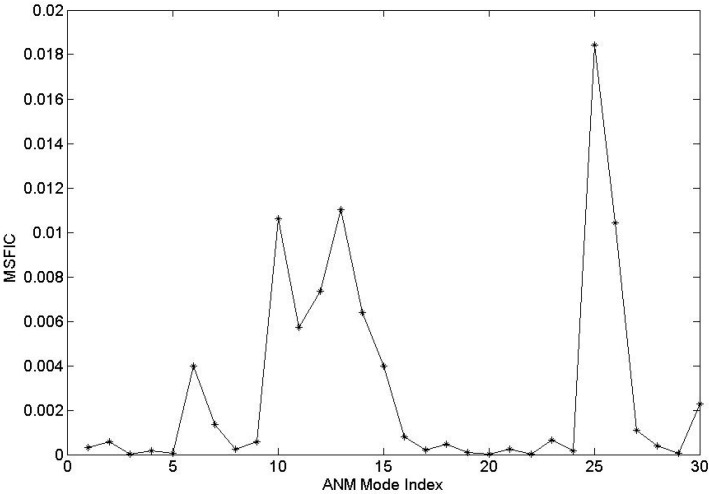
The calculated mean-square fluctuation of the internal coordinate (MSFIC) values for the first 30 slowest ANM modes. From this figure, it is found that the MSFIC values for modes 10, 13, 25 and 26 are relatively large.

In our method, the mean-square fluctuation of the internal coordinate (MSFIC) for each normal mode was calculated by using Equation (14) in the Methods section to evaluate the contribution of each mode to the channel-gating function of the system. It is believed that the low-frequency normal modes correspond to the large-scale collective motions, which are usually relevant to protein function. Therefore, in this work, we only analyze the first 30 slowest normal modes. The MSFIC values for these 30 modes were calculated and shown in Figure 2. From this figure, it is found that the MSFIC values of modes 10, 13, 25 and 26 are relative large, which implies that these modes are mainly responsible for the channel-gating motion of BtuCD–BtuF. While the MSFIC values for modes 1–5, 7–9, 16–24 and 27–29 are almost zero, which indicate that these modes are not related to the channel-gating function of the system. Modes 6, 11, 12, 14, 15 and 30 have small MSFIC values, implying that they moderately contribute to the protein functional motion.

**Figure 3:**
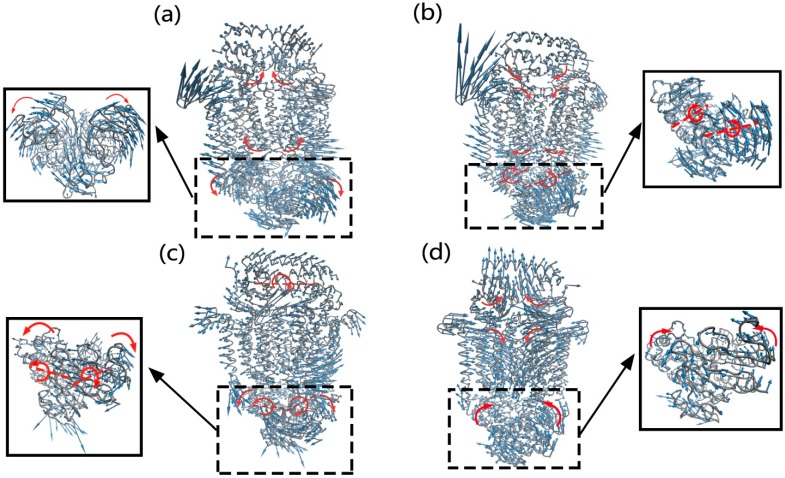
The motions of modes 10 (**a**), 13 (**b**), 25 (**c**) and 26 (**d**), which are relevant to the channel-gating function of the transporter. The close-up views of the NBDs for all the modes are also displayed. In this figure, the structure of the transporter is displayed in gray tube, and the amplitude and direction of the motions are denoted by the length and direction of the blue arrows. The red arrows and dotted lines respectively represent the motion direction and axis of the subdomains of the protein.

Then, the four normal modes with relative large MSFIC values were further analyzed in detail. In mode 10, as shown in Figure 3a and Movie S1 in the Supplementary Information, the two monomers of NBDs undergo a tweezers-like motion, where the C-terminal part of NBDs as the pivot and the N-terminal part as the tips. During the tweezers-like motion, the two NBD monomers move relatively as rigid bodies, which results in the increase of the distance between these two monomers and induces the opening of ATP binding pocket. The tweezers-like motion of NBDs also drives the two coupling helices, which connect NBDs and TMDs, to move apart. Through the connecting helices, the conformational motions in NBDs are transmitted to TMDs. The two monomers of TMDs perform a hinge bending motion, where the bending hinge is located at the middle region of the TMD dimer interface. The hinge bending motion of TMDs causes the opening of the cytoplasmic gate and slightly closing of the periplasmic gate of the substrate transport channel. The opening of the cytoplasmic gate enables the vitamin B12 to be squeezed out of the cavity in TMDs and translocated into the cytoplasm. Along this mode, the two lobes of BtuF bend inward slightly, which results in the closing of the substrate-binding cleft. It is also found that the conformational motion of BtuF is coupled with the channel-gating motion of TMDs through their connecting interface.

In mode 13, the two monomers of NBDs rotate reversely around their axes, respectively, where the rotation axis passes through the interface between the RecA-like subdomain and helical subdomain in each NBD monomer, as shown in Figure 3b and Movie S2 in the Supplementary Information. The rotation motions of these two monomers are symmetric with respect to the two-fold axis of NBDs, which results in the opening of the NBD dimer at the TMD side. Molecular dynamics (MD) simulation studies also found that the NBD dimer experiences rotation motions during the transport cycle [34,35]. The large-scale motions of NBDs also cause the connecting helices at the interface of NBDs and TMDs to move apart. For TMDs, the motion of the two monomers mainly appears as a hinge bending motion that is similar to mode 10, which results in the opening of the cytoplasmic channel gate and slightly closing of the periplasmic channel gate. The motion in TMDs is coupled with the collective motion of NBDs through the TMDs–NBDs connecting helices. These motions in TMDs and NBDs drive the conformational transition of the transporter from the nucleotide-bound intermediate state to the “inward-facing” state, which enable the vitamin B12 to be squeezed out of the substrate cavity in TMDs and translocated into the cell. For the vitamin B12 binding protein BtuF, the two lobes of the protein move towards each other, but the motion amplitude is very small. These results indicate that there exist long-range allosteric couplings among the motion of BtuF, the large-scale rotation motion of NBDs and the channel-gating motion of TMDs.

In mode 25, the two monomers of NBD undergo a tweezers-like motion, along with a reverse rotation around their respective axes passing through the interface of the two subdomains within each NBD monomer, as shown in Figure 3c and Movie S3 in the Supplementary Information. During the motion these two monomers move relatively as rigid bodies, and the motions of these two monomers are asymmetrical, where the motion amplitude of one monomer is distinctly larger than the other. The tweezers-like motion of NBDs causes the two NBD monomers to move apart at the TMD side, which induces the opening of ATP binding pocket. The conformational change of NBDs also drives the two NBDs–TMDs connecting helices to swing away from each other. Through the connecting helices, the movements in NBDs are transmitted to TMDs, which trigger a hinge bending motion for the two TMD monomers. The bending hinge is located at the periplasmic side of the TMD dimer interface. The motion amplitudes of these two monomers are also asymmetrical, which are coupled with the asymmetrical movements of NBDs. For BtuF, it rotates a whole. It should be noted that in this mode a BtuC loop is greatly extended as shown in Figure 3c and Movie S3. This dramatic conformational change is due to the simplification of the ANM model. In ANM, the number of residue connections is less for the loose structural parts of the protein, such as surface loops, and the fluctuation amplitude may be dramatically large.

Mode 26 represents a hinge bending motion of BtuF, as shown in Figure 3d and Movie S4 in the Supplementary Information. The two lobes of BtuF move towards each other as rigid bodies, and the bending hinge is located at the middle region of the linking helices that connect these two lobes. The hinge bending motion results in the closing of the substrate-binding pocket of BtuF. The conformational motion of BtuF also drives the closing of the periplasmic channel gate and opening of the cytoplasmic gate in TMDs through the BtuF–TMDs interaction interface. For NBDs, the two monomers undergo a slight closing motion. From Figure 3d and Movie S4, it is found that in this motion mode the periplasmic loops of BtuC are inserted into the vitamin B12 binding pocket of BtuF. Experimental studies have indicated that the insertion of periplasmic BtuC loops into the B12 binding pocket abrogates the binding affinity of BtuF for vitamin B12 and squeezes the B12 out of the binding pocket [13,14]. For the homogenous maltose transporter MalFGK_2_, the insertion of periplasmic MalG loops into the maltose-binding pocket of the maltose-binding protein was also observed [36].

The above results indicate that not all the low-frequency normal modes are relevant to the channel-gating function of BtuCD–BtuF. For the first 30 slowest normal modes, only four modes distinctly contribute to the channel-gating function of the protein. Our proposed method can evaluate the contribution of each normal mode to a specific function of the protein and then effectively identify the functional related modes. In addition, our analysis results show that for the vitamin B12 transport system, the motions in TMDs is allosterically coupled with the domain motions of NBDs and BtuF. The performance of the transporter function involves long-range communications between different domains in the protein.

### 2.2. The Motions of BtuCD–BtuF Relevant to the Channel-Gating Function

In our method, the normalized cross-correlation between the displacement of the internal coordinate and the movement of each residue in the protein was calculated according to Equation (15) in the Methods section to reveal the functional motions of BtuCD–BtuF. In our calculation, the first 30 slowest normal modes were added up according to their contributions to the channel-gating of the transporter. The modes that are related to protein channel-gating function will have large contributions to the value of the normalized cross-correlation, whereas the non-function-related normal modes will be excluded from the contribution to the cross-correlation value automatically.

**Figure 4:**
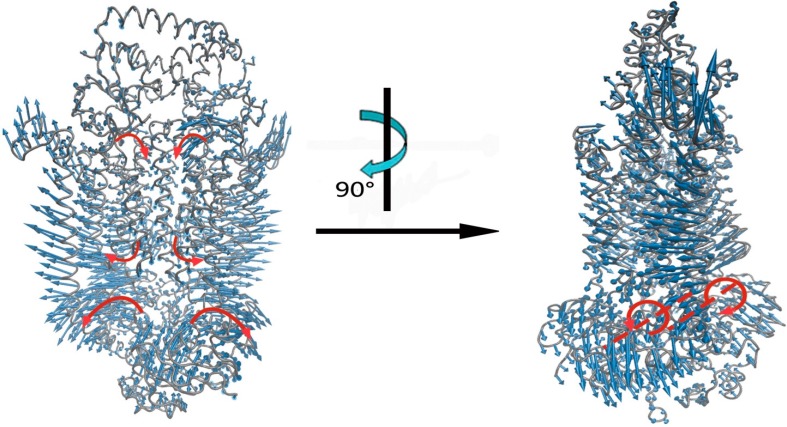
The functional motion that contributes to the channel-gating of BtuCD–BtuF. The functional motion was obtained by adding up the first 30 slowest normal modes of the system according to their contributions to the channel-gating of the transporter. In this figure, the structure of the protein is displayed in gray tube, and the amplitude and direction of the functional motion are denoted by the length and direction of the blue arrows. The red arrows and dotted lines respectively represent the motion direction and axis of the subdomains of the system.

The calculation result is shown in Figure 4 and Movie S5 in the Supplementary Information. It is found that the two monomers of NBDs mainly appear as a tweezers-like motion, in which the C-terminal region of NBDs serves as the pivot and the N-terminal part serves as the tips. Accompanied with the tweezers-like motion, these two NBD monomers also simultaneously undergo a slight rotation around their respective axes. The rotation directions for these two monomers are reverse, and the rotation axis for each NMD monomer passes through the interface of the two subdomains in the monomer. These two NBD monomers move as rigid body, and their motions are symmetric relative to the two-fold axis of NBDs. The collective motions of NBDs result in the two monomers to move apart from each other at the TMDs side, and cause the opening of the ATP binding pocket. Many studies have showed that the tweezers-like motion, along with reverse rotations, is a common functional motion mode of NBDs from various ABC transporters [33,35,37,38,39,40,41,42,43,44,45]. It is also found that in each NBD monomer the motion amplitude of the helical subdomain is distinctly larger than that of the RecA subdomain, which is well consistent with other experimental and computational studies [33,35,37,38,39,40,41,42,43,44,45]. The large-scale motion of NBDs also drives the NBDs–TMDs connecting helices to move away from each other. Through the connecting helices, the conformational movements of NBDs are coupled with the motions in TMDs. Experimental and computational studies also revealed that the connecting helices undergo large-scale swing motions during the substrate transport cycle, which play an important role for the conformational communication between NBDs and TMDs [34,35,40,42,44,45,46]. For TMDs, the two monomers perform a hinge bending motion, where the bending hinge is located at the periplasmic side of TMD dimer interface. Along this motion, the cytoplasmic sides of the TM helicases move away from each other, which results in the opening of the cytoplasmic channel gate, whereas the periplasmic sides of TMDs have a slightly tendency to contract the channel gate. Many studies also indicated that the hinge-bending motion is the intrinsic dynamical properties encoded in the structure of TMDs in BtuCD–BtuF [34,35,42,46]. The hinge-bending motion of TMDs drives the conformational transition from the intermediate state to the “inward-facing” state for the transporter. For the substrate binding protein BtuF, the two lobes of the protein undergo a slight open-closed motion, which is coupled with the hinge-bending motion of TMDs. Our calculation results show that for the functional motion of the transporter, the NBDs and the cytoplasmic side of TMDs undergo large conformational changes, whereas the movements in BtuF and the periplasmic side of TMDs are relatively small. This agrees well with the experimental observations [13]. Our calculation results also indicate that there exist long-range allosteric couplings between different domains of the transporter, which enables these domains to move cooperatively to carry out the transport function of the system. The above results are consistent with the experimental data [17].

### 2.3. The Key Residue Interactions that Control the Channel-Gating Motion of BtuCD–BtuF

**Figure 5:**
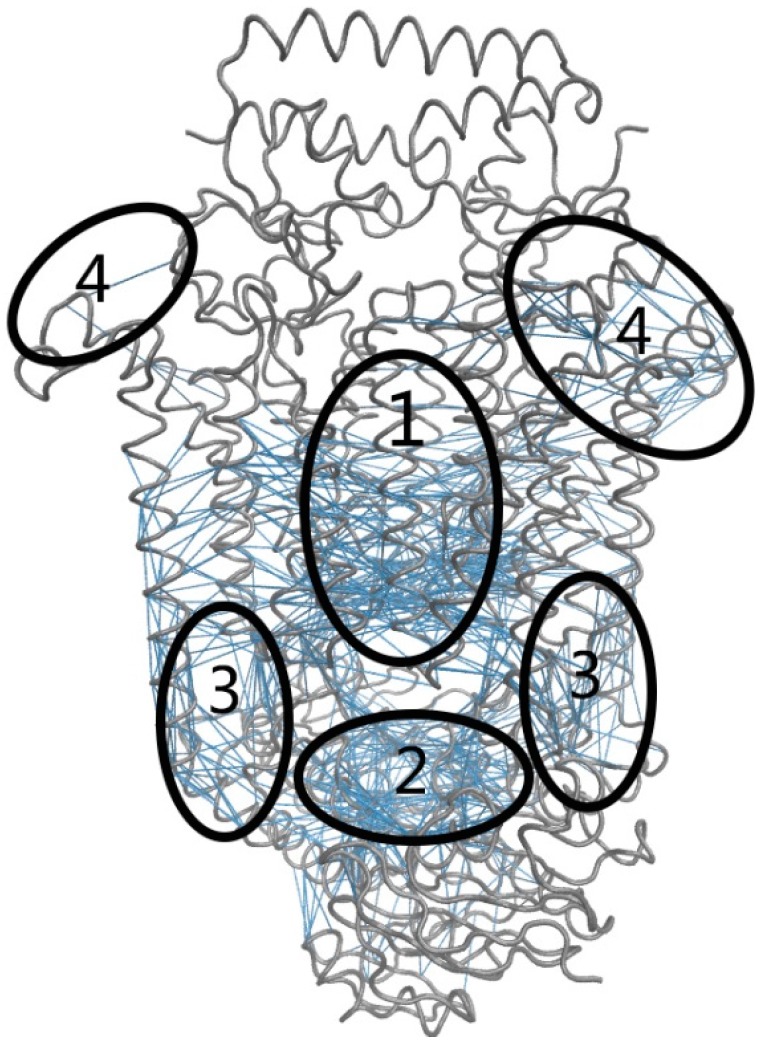
The functionally important residue interactions predicted by the perturbation method. The structure of BtuCD–BtuF is displayed in gray tube and the predicted key residue interactions are denoted by blue lines. According to their locations on protein structure, these key residue interactions are grouped into four regions that are denoted by ellipses and the numbers 1–4 in the figure.

In the present work, the functionally key residues were identified using a perturbation method, in which the residue interaction whose perturbation largely influences the fluctuation along the internal coordinate was considered as the key residue interaction. In this method, each residue interaction represented by a spring was perturbed, and then the change of the mean-square fluctuation of the internal coordinate δ((∆*r*)^2^) in response to the perturbation was calculated based on Equation (18) provided in the Methods Section. Considering that the low-frequency normal modes usually correspond to the collective functional motions, only the first 30 slowest normal modes were considered in our calculation. The residue interactions with relative large δ((∆*r*)^2^) values were identified as the key residue interactions responsible for the channel-gating motions of the transporter. Figure 5 displays the residue interactions with the first 5 percent largest δ((∆*r*)^2^) values. According to their locations on the structure of BtuCD–BtuF, these key residue interactions were grouped into four regions.

Group 1 represents the interactions between the transmembrane (TM) helices around the substrate transport channel in TMDs, as shown in Figure 5. These interactions control the relative motions of the TM helices. As discussed in the above section, the channel-gating function of the transporter is achieved by the hinge-bending motions of the TM helices. The residue interactions in Group 1 control the opening and closing of the cytoplasmic channel gate, and thus play a dominant role in the conformational transitions of the transporter from the intermediate state to the “inward facing” state. Experimental studies also revealed that the residues in the TM helices forming the substrate transport channel play crucial roles for the function of the transporter [47].

Group 2 is located at the NBD dimer interface, as shown in Figure 5. According to the discussion in the above section, during the substrate transport cycle the two monomers of NBDs undergo a tweezers-like motion, which induces the opening and closing of the ATP binding pocket. The inter-subunit interactions in Group 2 control the relative movements of the tips of the tweezers, and thus these interactions are crucial for the functional motions of NBDs, as well as the binding and hydrolysis of ATP. Our NMA calculation results also indicate that the tweezers-like motion of NBDs is significantly coupled with the channel-gating motion of TMDs, therefore, the residue interactions in Group 2 impact on the channel-gating function of the transporter through an allosteric mechanism. Experimental studies also found that point mutations in ATPase sites of NBDs significantly reduce the ATP hydrolysis and vitamin B12 transport activity [48].

Group 3 is located around the two coupling helices that connect NBDs and TMDs as shown in Figure 5. The analysis of the functional motions in the above section has revealed that coupled with the channel-gating motion of the transporter, the two NBDs–TMDs connecting helices swing back and forth with large scales. Through these two connecting helices, the movements of the two NBD monomers can transmit to TMDs, which then induce the opening and closing of the channel gate. Therefore, the NBDs–TMDs connecting helices mediate the coupling of the functional motions between NBDs and TMDs. The connecting helices exhibit a high degree of sequence and architecture homology among different ABC transporters, which implies a common coupling mechanism between NBDs and TMDs [49,50,51]. The functional importance of the connecting helices has been proved by many mutagenesis studies, in which the residue substitutions in this region impair the transport function of several ABC transporters [52,53,54].

Group 4 is located at the interface between BtuCD and BtuF as shown in Figure 5. It has been accepted that the substrate transport cycle is initiated by the docking of BtuF to the periplasmic side of BtuCD [13,55]. There exists a long-range allosteric communication between BtuF and BtuCD [17]. Our NMA calculation results also show that the open-closed motion of BtuF is coupled with the channel-gating motion of BtuCD. The residue interactions in Group 4 mediate the coupled inter-domain motions between BtuF and BtuCD. Experimental studies have showed that BtuF binds BtuCD with unusually high affinity [56], and the mutations of the residues at the BtuF-BtuCD interaction interface resulted in a considerable loss of the transport activity of the transporter, which indicates that the interactions between BtuF and BtuCD are functionally significant [57].

## 3. Experimental Section

### 3.1. Anisotropic Network Model (ANM)

In ANM (Anisotropic Network Model), a protein is considered as an elastic network, in which each residue is simplified as a point represented by its *C*_α_ atom. If the distance of two residue *C*_α_ atoms is less than the cutoff value (12.0 Å is adopted in this work), a spring is connected between them. All the springs are assumed to have the same force constant. In this model, the potential energy of the system can be written as [21,58]

(1) $$\text{V}=\frac{1}{2}\text{γ}\left\{\Delta {\vec{R}}^{T}\right\}H\left\{\Delta \vec{R}\right\}$$

where, γ is the force constant of the springs in the model; $\left\{\Delta \vec{R}\right\}$ is the 3N-dimensional column vector of the residue fluctuation, *N* is the number of residues in the protein; the superscript *T* represents the transpose of the column vector; and *H* is the Hessian matrix, which is expressed as

(2) $$H=\left[\begin{array}{cccc} H_{11} & H_{12} & \cdots & H_{1N}\\ H_{21} & H_{22} & \cdots & H_{2N}\\ \vdots & \vdots & \vdots & \vdots \\ H_{N1} & H_{N2} & \cdots & H_{NN} \end{array}\right]$$

where *H*_ij_ is a dimensional sub-matrix, whose elements are given as

(3) $$H_{ij}=\left[\begin{array}{ccc} \frac{\partial^{2}V}{\partial x_{i}\partial x_{j}} & \frac{\partial^{2}V}{\partial x_{i}\partial y_{j}} & \frac{\partial^{2}V}{\partial x_{i}\partial z_{j}}\\ \frac{\partial^{2}V}{\partial y_{i}\partial x_{j}} & \frac{\partial^{2}V}{\partial y_{i}\partial y_{j}} & \frac{\partial^{2}V}{\partial y_{i}\partial z_{j}}\\ \frac{\partial^{2}V}{\partial z_{i}\partial x_{j}} & \frac{\partial^{2}V}{\partial z_{i}\partial y_{j}} & \frac{\partial^{2}V}{\partial z_{i}\partial z_{j}} \end{array}\right]$$

The Hessian matrix *H* can be decomposed as

(4) $$H=\text{UΛ}U^{T}$$

where *U* is the orthogonal matrix whose column *u*_i_ represents the eigenvector of *H*, and Λ is the diagonal matrix whose diagonal element λ*_i_* is the eigenvalue of *H*. There are six modes with zero eigenvalues, which correspond to three overall translational and three overall rotational motions of the protein. These eigenvectors with zero eigenvalue are removed from our analysis. The rest of the 3N-6 eigenvectors with non-zero eigenvalues represent the modes of motions within the protein structure. The low-frequency normal modes correspond to the large-scale collective motions, which are usually related to protein functional motions. While, the high-frequency normal modes are responsible for geometric irregularity in the local structure of proteins.

The mean-square fluctuation for the *i*^th^ residue can be calculated from the inverse of the Hessian matrix expressed as

(5) $$\langle {\left(\Delta {\vec{R}}_{i}\right)}^{2}\rangle =\langle {\left(\Delta X_{i}\right)}^{2}\rangle +\langle {\left(\Delta Y_{i}\right)}^{2}\rangle +\langle {\left(\Delta Z_{i}\right)}^{2}\rangle =\frac{k_{B}T}{\text{γ}}\left(H_{3i-2,3i-2}^{-1}+H_{3i-1,3i-1}^{-1}+H_{3i,3i}^{-1}\right)$$

The *B*-factor of the *i*^th^ residue can be calculated by

(6) $$B_{i}=\frac{8\pi^{2}}{3}\langle {\left(\Delta {\vec{R}}_{i}\right)}^{2}\rangle =\frac{8{\text{π}}^{2}k_{B}T}{3\text{γ}}\left(H_{3i-2,3i-2}^{-1}+H_{3i-1,3i-1}^{-1}+H_{3i,3i}^{-1}\right)$$

in this work, the calculated *B*-factors were compared with the experimental data to determine the value of the spring constant γ.

The cross-correlation between *i*^th^ and *j*^th^ residues can be obtained by

(7) $$\langle \Delta {\vec{R}}_{i}\cdot \Delta {\vec{R}}_{j}\rangle =\langle \Delta X_{i}\cdot \Delta X_{j}\rangle +\langle \Delta Y_{i}\cdot \Delta Y_{j}\rangle +\langle \Delta Z_{i}\cdot \Delta Z_{j}\rangle =\frac{k_{B}T}{\text{γ}}\left(H_{3i-2,3i-2}^{-1}+H_{3i-1,3i-1}^{-1}+H_{3i,3i}^{-1}\right)$$

### 3.2. The Internal Coordinate Related to the Channel-Gating Function of BtuCD-F

In the structure of BtuCD–BtuF, TMD dimer forms the substrate transport channel whose cytoplasmic gate controls the translocation of the substrate into the cell. During the substrate transport cycle, BtuCD–BtuF undergoes large-scale domain movements, which results in the open–closed motions of the channel gate. In the present work, the extent of opening of the cytoplasmic gate was chosen as an internal coordinate that is relevant to the channel-gating function of the transporter. The extent of opening of the gate is defined as the average distance between residues located on the opposite sides of the cytoplasmic gate. In the computation, four residues around the cytoplasmic gate in each monomer were chosen, *i.e.*, Ser143, Arg144, Leu147 and Ala148. Then the average distance between the residue pairs formed by these residues from the two monomers, *i.e.*, Ser143–Ser143, Arg144–Arg144, Leu147–Leu147 and Ala148–Ala148, was calculated as the functional internal coordinate. The motions along this internal coordinate are considered to be responsible for the channel-gating function of the transporter.

The distance between two residues located on the opposite sides of the cytoplasmic gate can be calculated as

(8) $$r_{ij}={\left[{\left(x_{i}-x_{j}\right)}^{2}+{\left(y_{i}-y_{j}\right)}^{2}+{\left(z_{i}-z_{j}\right)}^{2}\right]}^{\frac{1}{2}}$$

where, *x*_i_, *y*_i_, *z*_i_, and *x*_j_, *y*_j_, *z*_j_ represent the position of the *i*^th^ and *j*^th^ residues, respectively.

The deviation of the distance in response to the displacements of the *i*^th^ and *j*^th^ residues can be expressed as

(9) $$\Delta r_{ij}=\frac{\partial r_{ij}}{\partial x_{i}}\Delta x_{i}+\frac{\partial r_{ij}}{\partial y_{i}}\Delta y_{i}+\frac{\partial r_{ij}}{\partial z_{i}}\Delta z_{i}+\frac{\partial r_{ij}}{\partial x_{j}}\Delta x_{j}+\frac{\partial r_{ij}}{\partial y_{j}}\Delta y_{j}+\frac{\partial r_{ij}}{\partial z_{j}}\Delta z_{j}$$

Then, the displacement of the average distance for the four residue pairs located on the opposite sides of the cytoplasmic gate can be computed as

(10) $$\Delta \text{r}=\frac{\Delta r_{1}+\Delta r_{2}+\Delta r_{3}+\Delta r_{4}}{4}$$

where ∆*r*_1_, ∆*r*_2_, ∆*r*_3_, ∆*r*_4_ are, respectively, the displacements in the distance for each of the four residue pairs, which can be calculated by Equation (9).

To facilitate the analysis, the above function-related internal coordinate was introduced as one of the axes of the coordinate space. In order to maintain the completeness and non-redundancy of the coordinate space, one of the Cartesian coordinates, assumed as the Cartesian coordinate, should be replaced by this internal coordinate. Then, the conventional Cartesian coordinate space was transformed into the internal/Cartesian space with linear approximation expressed by matrix notation as

(11) {ΔrΔR}=A{ΔR}

where {∆*r*/∆*R*} is the 3N-dimensional column vector of the internal/Cartesian space, which is composed of the introduced one internal coordinate and 3N-1 Cartesian coordinates; {∆*R*} is the 3N-dimensional Cartesian displacements; and *A* is the transformation matrix.

### 3.3. Methods to Analyze the Function-Related Motions and Identify the Functionally Important Residue Interactions

In order to perform normal mode analysis (NMA) in this new internal/Cartesian coordinate space, the Hessian matrix in the conventional Cartesian space can be transformed into that in the internal/Cartesian space by

(12) $$H\prime ={\left(A^{-1}\right)}^{T}HA^{-1}$$

where *H*′ is the Hessian matrix in the conventional Cartesian space and *H*′ is the Hessian matrix in the internal/Cartesian space. Then, the Hessian matrix *H*′ can be decomposed as

(13) $$H\prime =U\prime \text{Λ}\prime {\left(U\prime \right)}^{T}$$

where *U*′ is the orthogonal matrix whose column $U_{i}^{\prime }$ epresents the eigenvector of *H*′, and Λ′ is the diagonal matrix whose diagonal element ${\text{λ}}_{i}^{\prime }$ is the eigenvalue of *H*′. The first six eigenvectors with zero eigenvalue are removed from our analysis, and the remaining nonzero eigenvectors are considered in our analysis.

The following analysis methods were applied to explore the function-related motion modes and identify the functionally important residue interactions in the studied transporter.

#### 3.3.1. The Mean-Square Fluctuation of the Internal Coordinate for Each Normal Mode

The mean-square fluctuation of the internal coordinate for each normal mode can be calculated by

(14) $${\langle {\left(\text{Δ}r\right)}^{2}\rangle }_{k}=\frac{k_{B}T}{\gamma }{\lambda^{\prime }}_{k}^{-1}{\left[u_{k}^{\prime }\right]}_{m}{\left[u_{k}^{\prime }\right]}_{m}$$

where *k*_B_ is the Boltzmann constant; *T* is the absolute temperature; γ is the spring constant; and ∆*r* represents the internal coordinate, which is the *m*^th^ element in the column vector of the angular/Cartesian coordinate displacements. In the present work, this quantity is used to evaluate the contribution of each normal mode to the channel-gating motion of the protein.

#### 3.3.2. The Cross-Correlation between the Displacement of the Inter-Distance and the Movement of Each Residue in the Transporter

The cross-correlation between the displacement of the inter-distance and the movement of the *i*^th^ residue can be calculated by

(15) $$\langle \Delta r\Delta \vec{R_{i}}\rangle =\langle \Delta r\Delta x_{i}\rangle \vec{e_{x}}+\langle \Delta r\Delta y_{i}\rangle \vec{e_{y}}+\langle \Delta r\Delta z_{i}\rangle \vec{e_{z}}=\frac{k_{b}T}{\gamma }\left(H\prime_{m,3i-2}^{-1}\vec{e_{x}}+H\prime_{m,3i-1}^{-1}\vec{e_{y}}+H\prime_{m,3i}^{-1}\vec{e_{z}}\right)$$

where ${\vec{e}}_{x},{\vec{e}}_{y},{\vec{e}}_{z}$ are the unit vectors in the directions of *x*, *y* and *z* axes, respectively. In this study, this quantity is used to reveal the amplitudes and directions of the functional motions for the protein.

#### 3.3.3. The Perturbation Method to Identify Functionally Key Residue Interactions

Similar to the method proposed by Zheng and Brooks [27], in the perturbation approach, each spring was perturbed and the residue interactions whose perturbation has large influence on the mean-square fluctuation of the internal coordinate were identified as the functionally key residue interactions.

If the force constant of the spring γ*_ij_* connecting the *i*^th^ and *j*^th^ residues is perturbed, the change of the interaction energy in response to the perturbation can be computed by

(16) $$\delta E=\frac{1}{2}{\text{δγ}}_{ij}{\left(d_{ij}-d_{ij}^{0}\right)}^{2}$$

Then, the change of the Hessian matrix elements can be calculated by

(17) $$\text{δ}H_{k\text{α},l\text{β}}^{\prime }=\frac{\partial^{2}\delta E}{\partial x_{k\alpha }\partial x_{l\beta }}$$

here, *k* and *l* represent the *k*^th^ and *l*^th^ residues, respectively, and α, β = 1, 2, 3 denote *x*, *y*, *z* components of the coordinate, respectively. According to the change of the Hessian matrix, the change in the mean-square fluctuation of the internal coordinate in response to the perturbation can be written as

(18) $$\text{δ}\langle {\left(\Delta r\right)}^{2}\rangle =-{\left({H^{\prime }}^{-1}\delta H^{\prime }{H^{\prime }}^{-1}\right)}_{mm}$$

where m represents the internal coordinate, which is the $m^{th}$element in the column vector of the angular/Cartesian coordinate displacements. In the present work, the functionally key residue interactions are identified as those with relative larger δ((∆*r*)^2^) values.

## 4. Conclusions

BtuCD–BtuF from *Escherichia coli* is an ABC transporter that carries substrate vitamin B12 across cellular membranes using the energy of ATP hydrolysis. During the substrate transport cycle, the translocation channel formed by TMDs undergoes an open–closed motion. In the present work, the structure-encoded conformational motions and the key residue interactions responsible for the channel-gating function of the transporter were analyzed using a new ENM-based method. In our method, the extent of opening of the cytoplasmic gate was introduced as an internal coordinate that is relevant to the channel-gating function of the protein, and the conventional Cartesian coordinate space was transformed into the internal/Cartesian space, in which the introduced internal coordinate serves as one of the coordinate axes. NMA of ENM in this new internal/Cartesian coordinate space was carried out to analyze the functional motions related to the channel-gating of the transporter. Besides that, the functionally important residue interactions that allosterically control the channel-gating motion of the transporter were identified by using a perturbation method.

Our calculation results show that for the first 36 slowest normal modes, only four modes distinctly responsible for the channel-gating function of BtuCD–BtuF. For NBDs, the functional motion mainly appears as a tweezers-like motion along with a slight rotation. TMDs undergo a hinge-bending motion, which results in opening of the cytoplasmic channel gate. The motion of TMDs results in the vitamin B12 to be squeezed out of the substrate cavity and ejected into the cytoplasm. The vitamin B12 binding protein BtuF mainly exhibits a slight open–closed movement for its two lobes. It is found that the movements of NBDs and the cytoplasmic side of TMDs are relative large, whereas the motions in BtuF and the periplasmic side of TMDs are very small. Our results also indicate that the functional motions of TMDs are allosterically coupled with the large-scale motions in NBDs and BtuF through the inter-domain interfaces. Besides that, our studies found that the functionally important residue interactions that control the channel-gating motion of the transporter are mainly located at the following regions: the interactions between the TM helices around the substrate transport channel in TMDs, the NBD dimer interface, the two coupling helices region that connect NBDs and TMDs, and the interface between BtuCD and BtuF. Our calculation results are consistent with the available experimental observations. The present study is helpful for our understanding of the vitamin B12 transport mechanism of BtuCD–BtuF.

## Supplementary Materials

Supplementary materials can be found at http://www.mdpi.com/1422-0067/16/08/17933/s1.
