# Supplementary figures and images for: Conformational Motions and Functionally Key Residues for Vitamin B12 Transporter BtuCD–BtuF Revealed by Elastic Network Model with a Function-Related Internal Coordinate
Source: Int J Mol Sci. 2015 Aug 4;16(8):17933–51. doi: 10.3390/ijms160817933 (PMC4581229; doi:10.3390/ijms160817933)

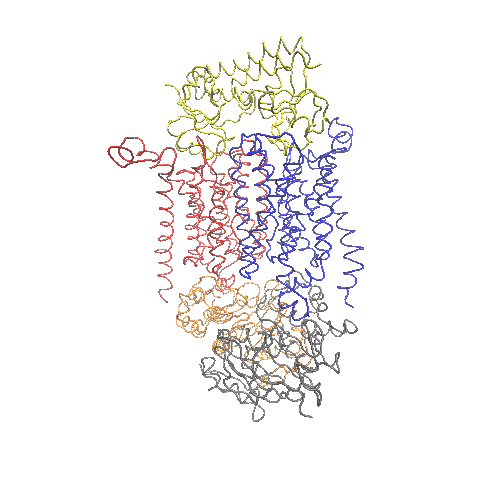

Supplement: Supplementary file 1 [file ijms-16-17933-s001.zip › Supplementary Files/Movie S1.gif]

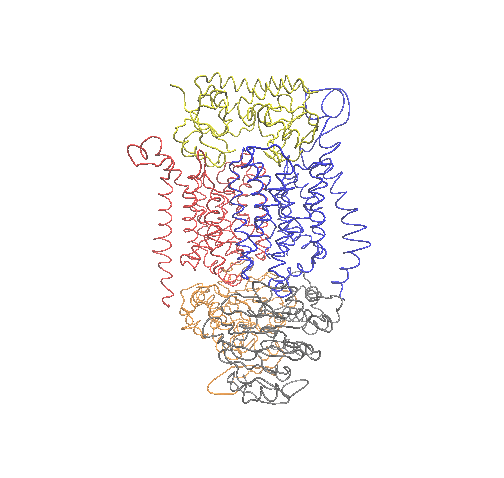

Supplement: Supplementary file 1 [file ijms-16-17933-s001.zip › Supplementary Files/Movie S2.gif]

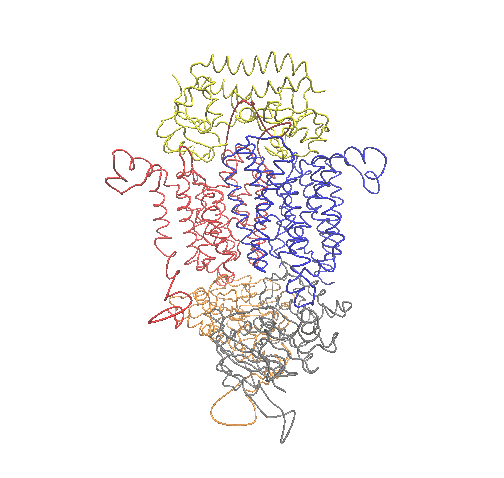

Supplement: Supplementary file 1 [file ijms-16-17933-s001.zip › Supplementary Files/Movie S3.gif]

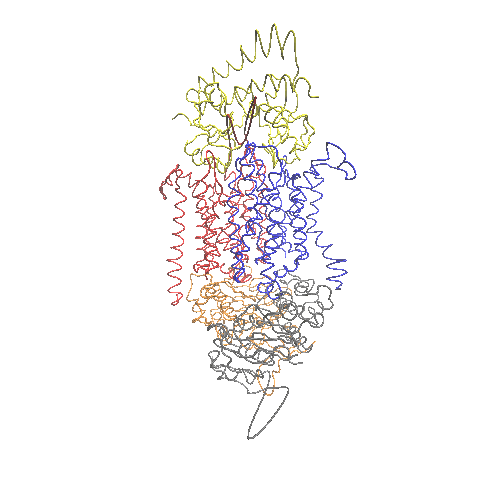

Supplement: Supplementary file 1 [file ijms-16-17933-s001.zip › Supplementary Files/Movie S4.gif]

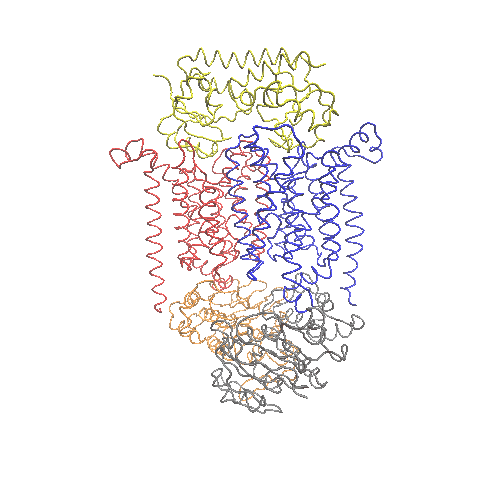

Supplement: Supplementary file 1 [file ijms-16-17933-s001.zip › Supplementary Files/Movie S5.gif]
